# Postnatal lethality and chondrodysplasia in mice lacking both chondroitin sulfate *N*-acetylgalactosaminyltransferase-1 and -2

**DOI:** 10.1371/journal.pone.0190333

**Published:** 2017-12-29

**Authors:** Miki Shimbo, Riku Suzuki, Sayaka Fuseya, Takashi Sato, Katsue Kiyohara, Kozue Hagiwara, Risa Okada, Hiromasa Wakui, Yuki Tsunakawa, Hideto Watanabe, Koji Kimata, Hisashi Narimatsu, Takashi Kudo, Satoru Takahashi

**Affiliations:** 1 Department of Anatomy and Embryology, Faculty of Medicine, University of Tsukuba, Tsukuba, Ibaraki, Japan; 2 Doctoral Program in Biomedical Sciences, Graduate School of Comprehensive Human Sciences, University of Tsukuba, Tsukuba, Ibaraki, Japan; 3 Ph.D. Program in Human Biology, School of Integrative and Global Majors, University of Tsukuba, Tsukuba, Ibaraki, Japan; 4 Master’s Program in Medical Sciences, Graduate School of Comprehensive Human Sciences, University of Tsukuba, Tsukuba, Ibaraki, Japan; 5 Glycoscience and Glycotechnology Research Group, Biotechnology Research Institute for Drug Discovery, National Institute of Advanced Industrial Science and Technology (AIST), Tsukuba, Ibaraki, Japan; 6 Institute for Molecular Science of Medicine, Aichi, Japan; 7 Multidisciplinary Pain Center, Aichi Medical University, Aichi, Japan; 8 Laboratory Animal Resource Center (LARC), University of Tsukuba, Tsukuba, Ibaraki, Japan; 9 Transborder Medical Research Center, Faculty of Medicine, University of Tsukuba, Tsukuba, Ibaraki, Japan; Kyungpook National University School of Medicine, REPUBLIC OF KOREA

## Abstract

Chondroitin sulfate (CS) is a sulfated glycosaminoglycan (GAG) chain. In cartilage, CS plays important roles as the main component of the extracellular matrix (ECM), existing as side chains of the major cartilage proteoglycan, aggrecan. Six glycosyltransferases are known to coordinately synthesize the backbone structure of CS; however, their *in vivo* synthetic mechanism remains unknown. Previous studies have suggested that two glycosyltransferases, Csgalnact1 (t1) and Csgalnact2 (t2), are critical for initiation of CS synthesis *in vitro*. Indeed, *t1* single knockout mice (*t1* KO) exhibit slight dwarfism and a reduction in CS content in cartilage compared with wild-type (WT) mice. To reveal the synergetic roles of t1 and t2 in CS synthesis *in vivo*, we generated systemic single and double knockout (DKO) mice and cartilage-specific *t1* and *t2* double knockout (Col2-DKO) mice. DKO mice exhibited postnatal lethality, whereas *t2* KO mice showed normal size and skeletal development. Col2-DKO mice survived to adulthood and showed severe dwarfism compared with *t1* KO mice. Histological analysis of epiphyseal cartilage from Col2-DKO mice revealed disrupted endochondral ossification, characterized by drastic GAG reduction in the ECM. Moreover, DKO cartilage had reduced chondrocyte proliferation and an increased number of apoptotic chondrocytes compared with WT cartilage. Conversely, primary chondrocyte cultures from Col2-DKO knee cartilage had the same proliferation rate as WT chondrocytes and low GAG expression levels, indicating that the chondrocytes themselves had an intact proliferative ability. Quantitative RT-PCR analysis of E18.5 cartilage showed that the expression levels of *Col2a*1 and *Ptch1* transcripts tended to decrease in DKO compared with those in WT mice. The CS content in DKO cartilage was decreased compared with that in *t1* KO cartilage but was not completely absent. These results suggest that aberrant ECM caused by CS reduction disrupted endochondral ossification. Overall, we propose that both t1 and t2 are necessary for CS synthesis and normal chondrocyte differentiation but are not sufficient for all CS synthesis in cartilage.

## Introduction

Chondroitin sulfate (CS) is a long-linear glycosaminoglycan (GAG) chain consisting of repeating sulfated disaccharide units of *N*-acetylgalactosamine (GalNAc) and glucuronic acid (GlcUA) that is covalently attached to core proteins via a linkage region to form a proteoglycan (PG). Depending on the type of core proteins, chondroitin sulfate proteoglycans (CSPGs) can be cell membrane-bound or part of the extracellular matrix (ECM), are particularly abundant in cartilage and the brain, and play an important role in development and homeostasis. Aggrecan is a large CSPG comprising approximately 100 CS chains and residing in the ECM. It contributes to water retention and the compression resistance of cartilage by aggregating with hyaluronan and link protein 1 [1]. In brain ECM, various CSPGs, including aggrecan, versican, and neurocan, form a structure called a perineuronal net that surrounds neuronal cell bodies to regulate their plasticity and activity [2–4]. Not only core proteins but also CS chains have indispensable roles in CSPG function. Among the functions, glycosylation prevents the core protein from degrading [5], and we previously reported that reduced CS chains accelerate core protein degradation of cartilage aggrecan [6]. Furthermore, CS shows diversity in sulfation patterning, exerting effects on cortical layer formation and axon guidance of retinal growth cones [7,8]. Although the mechanism of CS recognition by other proteins remains unknown, previous studies revealed that cytokines and growth factors, such as midkine and pleiotrophin [9], as well as receptors, including transmembrane protein tyrosine phosphatase (PTPσ) and contactin-1 [10–12], bind to CS chains.

**Fig 1:**
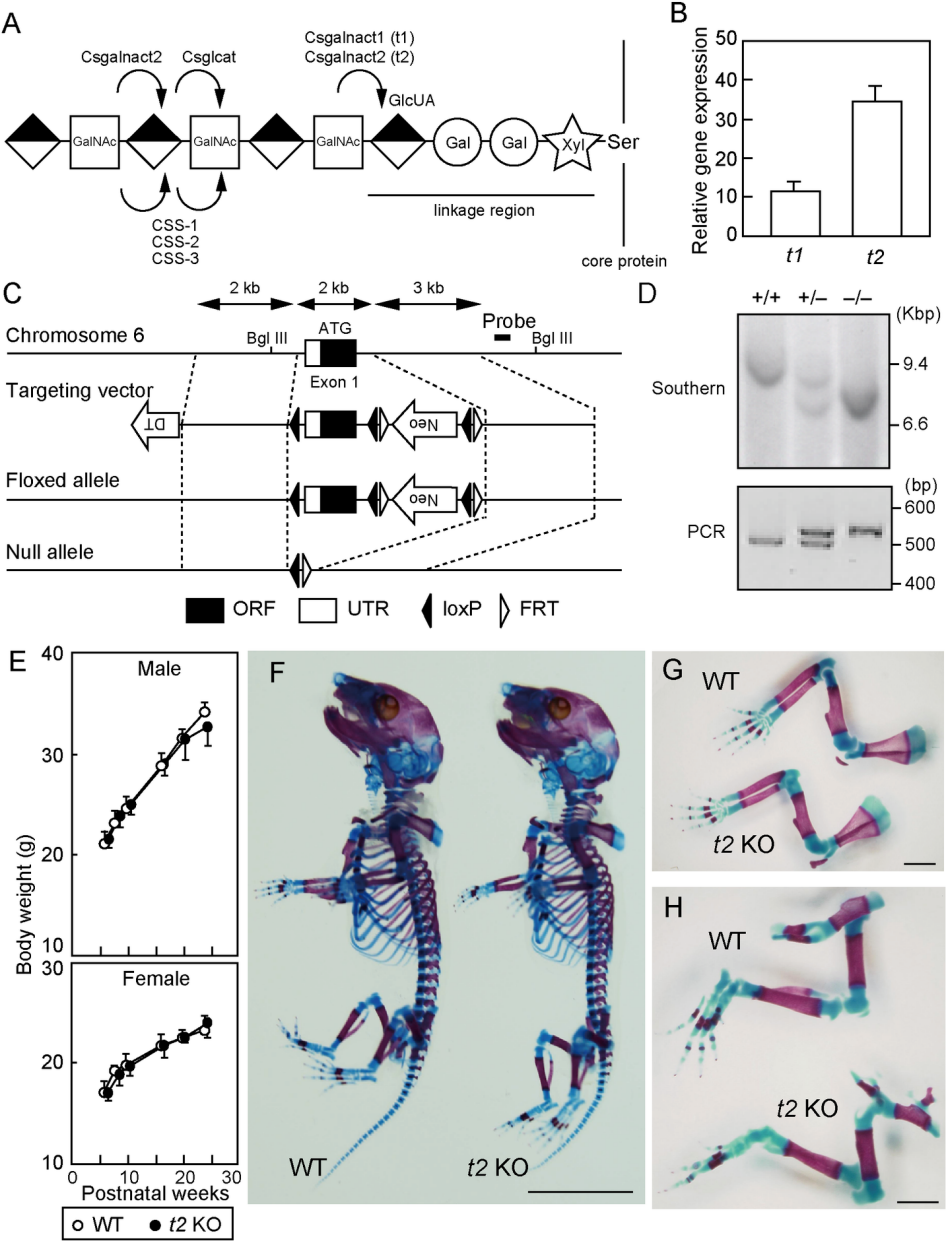
Phenotype of *t2* null mice. (A) Schematic showing the CS biosynthetic pathway and relevant glycosyltransferases. Arrows indicate the catalytic activity of each glycosyltransferase. Half-filled diamonds, open squares, open circles, and stars refer to GlcA, GalNAc, Gal, and Xyl, respectively. (B) Quantitative analysis of *t1* and *t2* gene transcription in humeral cartilage of WT mice (n = 3) using real-time RT-PCR. The expression of each transcript was normalized to that of β-actin. (C) Targeting strategy for conditional deletion of the *t2* gene. The exon containing the initiation codon and transmembrane domain was flanked by loxP elements. This region was deleted via mating with Ayu1-Cre mice, to generate systemic *t2* KO mice. Probe position for Southern hybridization is indicated by the bold line. (D) Southern blot analysis of *Bgl* II-digested genomic DNA and PCR-based genotyping of progeny from intercrossing heterozygotes. (E) Postnatal growth kinetics of WT mice (male, n = 3; female, n = 6) and *t2* KO littermates (male, n = 11; female, n = 5). *F*-*H*, Double whole body staining with Alizarin red and Alcian blue (*F*), upper (*G*) and lower limbs (*H*) of the WT and *t2* KO littermates at E18.5. Scale bars: 1 cm (*F*) and 1 mm (*G*, *H*).

CS biosynthesis is initiated by the transfer of GalNAc residues to the linkage region, which consists of tetrasaccharide units of GlcUA-β1,3-galactose (Gal)-β1,3-Gal-β1,4-xylose (Xyl) attached to the serine residues of the core proteins (Fig 1A). This triggers CS elongation by alternating addition of GalNAc and GlcUA residues, which is catalyzed by six glycosyltransferases in mammals. The CS glycosyltransferases are classified into three pairs based on their amino acid sequence similarity. The first pair is chondroitin sulfate synthase 1 (CSS1)/chondroitin synthase 1 (ChSy1) and chondroitin sulfate synthase 3 (CSS3)/chondroitin synthase 2 (ChSy2), which conduct dual glycosyltransferase activities of β1,3-glucuronyltransferase (β3GlcA-T) and β1,4-galactosaminyltransferase (β4GalNAc-T). The second pair is chondroitin sulfate glucuronyltransferase (CSGlcAT)/chondroitin synthase 3 (ChSy3) and chondroitin sulfate synthase 2 (CSS2)/chondroitin polymerizing factor (ChPF). CSS2 exhibits dual glycosyltransferase activities similar to CSS1 and CSS3, whereas CSGlcAT performs only β3GlcA-T activity. The third pair is chondroitin sulfate *N*-acetylgalactosaminyltransferase-1 (CSGalNAcT-1; t1) and -2 (CSGalNAcT-2; t2), which exhibit β4GalNAc-T activity only. The glycosyltransferase activities for CS biosynthesis can be categorized into two types; “initiation activity”, in which the first GalNAc is transferred to the linkage region, and “elongation activity” for GalNAc and GlcUA polymerization after initiation. The former four glycosyltransferases are thought to regulate elongation activity, whereas t1 and t2 are known to function in both initiation and elongation activity [13–19]. We previously reported that t1 exhibits stronger initiation activity than t2, indicating that t1 has a vital role in CS synthesis initiation [20].

Although the CS glycosyltransferase activities have been well studied *in vitro*, their function *in vivo* remains unknown. To examine this, CS glycosyltransferase knockout (KO) mice were generated and analyzed. *Css2* KO mice showed no morphological phenotypes; however, their CS chain length in cartilage was significantly shorter than that of wild-type (WT) mice [21], suggesting that Css2 has elongation activity *in vivo*, similar to that observed with *in vitro* studies. Conversely, *Css1* KO mice displayed skeletal phenotypes including abnormal digit patterning and endochondral ossification, and their CS sulfation patterns in cartilage were altered [21], suggesting that Css1 may regulate sulfation *in vivo*, which was not shown *in vitro*. With regard to this, *t1* KO mice also showed abnormal endochondral ossification, which resulted in slight dwarfism and impaired aggrecan metabolism [6,22]. Their CS showed no difference in chain length or sulfation patterns, but the number of CS chains decreased by approximately half. These results not only replicated the *in vitro* results of t1 initiation activity *in vivo* but also suggested the possibility that CS glycosyltransferases other than t1 have initiation activity, which raises the hypothesis that t1 and t2 both regulate the initiation activity *in vivo*.

In the current study, we generated and characterized *Csgalnact1*::*Csgalnact2* double KO (DKO) mice. DKO mice exhibited postnatal lethality due to respiratory failure and mild dwarfism and their cartilage showed more severe abnormality of endochondral ossification due to reduction of CS than cartilage from *t1* KO mice. These observations suggest that CS synthesis is not enough for t1 and t2 and the decreased CS in cartilage causes abnormal chondrocyte function.

## Materials and methods

### Animals

Mice were maintained under specific pathogen-free conditions at the Laboratory Animal Resource Center of the University of Tsukuba. All experiments were approved by the institutional animal care and use committees of the University of Tsukuba (No. 16–102 and 17–106) and were conducted according to related guidelines and the applicable laws of Japan. Mouse cages were placed in an air-conditioned room (average temperature; 24.1°C, average relative humidity; 42.8%RH) with a 12:12-h light-dark cycle. The mice were fed with CRF-1 (Oriental Yeast Co., Ltd., Tokyo, Japan) and given water *ad libitum*. The drinking water was autoclaved tap water. We checked the health condition of the mice every day, and unexpected deaths were very rare. The humane endpoints that we used were behavioral changes and 20% weight loss. All mice were euthanized via the inhalation of lethal doses of isoflurane and then subjected to dissection to collect tissue samples.

### Generation of mice with genetically modified *t1* and *t2* genes

The generation of *t1* KO mice and mice carrying the floxed *t1* gene was described in a previous report by our group [6]. To distinguish the *t1*^*+*^, *t1*^*-*^, and *t1*^*flox*^ alleles, the genotypes of the mice were confirmed via PCR using the following 3 primers: 5’-TAGATGAACTGTCCATCCTACAG-3’, 5’-GAGACGGCTCTCTTGCTTCCAAGG-3’, and 5’-CCTTTACTAAAATGGCGACCTGCC-3’.

To generate mice carrying a genetically modified *t2* gene, a targeting vector to disrupt the *t2* gene was constructed by ligating 3 PCR fragments into a conditional targeting vector cassette [6]. The constructed targeting vector was linearized via *Not*I digestion and transfected into C57BL/6J embryonic stem (ES) cells [23]. The resulting cells were subsequently selected in medium containing 1% G418 (Nacalai Tesque, Kyoto, Japan), and correct homologous recombination was confirmed through PCR and Southern hybridization (Fig 1D). Targeted ES cells were injected into ICR blastocysts to generate chimeric mice. Male mice chimeric for the targeted allele were mated with female ACTB-FLPe transgenic mice to remove the neomycin resistance cassette via excisional recombination of the Flp/FRT system, which generated a floxed *t2* allele (*t2*^*flox/+*^). To generate heterozygous exon 1-deleted mice (*t2*^*+/-*^), mice carrying the *t2*^*flox*^ allele were crossed with Ayu-1 Cre mice, a general deleter transgenic line expressing Cre recombinase, including in the germ line [24].

To generate chondrocyte-specific conditional *t1* and *t2* KO mice, *t1*^*flox/-*^::*t2*^*flox/-*^ mice were further crossed with transgenic mice expressing Cre recombinase under the collagen2a1 promoter (Col2-cre (B6;SJL-Tg(Col2a1-cre)1Bhr/J), Jackson Laboratory, Bar Harbor, ME, USA). The primers used for the genotyping of Col2-cre were 5’-GGACATGTTCAGGGATCGCCAGGCGT-3’ and 5’-GCATAACCAGTGAAACAGCATTGCTG-3’.

### Quantitative analysis of six CS glycosyltransferase transcripts using real-time RT-PCR

Total RNA was isolated from the humeral cartilage of embryonic day 18.5 (E18.5) embryos using Isogen (Nippon Gene, Tokyo, Japan), and cDNA templates were synthesized from the total RNA with a QuantiTect Reverse Transcription Kit (QIAGEN, Venlo, the Netherlands). The primers and probes selected from TaqMan Gene Expression Assays (Applied Biosystems, Foster City, CA, USA) and the primers used with the THUNDERBIRD SYBR qPCR system (Toyobo, Osaka, Japan) are listed in S3 Table. PCR products were continuously measured with a 7500 Fast Real-Time PCR System (Applied Biosystems). Relative transcript levels were normalized to the amount of the *β-actin* transcript in the same cDNA sample.

### Skeletal histology

The whole skeleton of E18.5 embryos was fixed in 95% ethanol and stained overnight in a solution containing Alcian blue 8GX (Sigma-Aldrich, St. Louis, MO, USA). Samples were placed in 1% KOH (vol/vol)/Alizarin red S (Sigma-Aldrich) for 2 h and then cleared with 2%-0.2% KOH (vol/vol)/20-80% glycerol for several days. To measure bone length, isolated humeri and tibiae were photographed with a digital microscope (VH-8000; KEYENCE, Osaka, Japan) and then measured with ImageJ software (freeware; National Institute of Health, USA).

### Histological and immunohistochemical analyses

Dissected mouse tibiae, tracheae, and lungs were fixed overnight in Mildform 10N (WAKO Pure Chemical Industries, Ltd. (WAKO), Osaka, Japan) at 4°C and then embedded in paraffin. Hard skeletal tissues of postnatal day 14 (P14) pups were decalcified in EDTA solution (pH 8.0) for 5 days before being embedded in paraffin. Four-micrometer-thick sections prepared from the lungs were subjected to HE staining, and longitudinal sections of the proximal tibial epiphysis and tracheal cartilage were stained with Safranin-O. To confirm the Safranin-O staining rate of CS among all GAG in cartilage, WT tibial sections were digested with 1 U/ml chondroitinase ABC (Seikagaku Corp., Tokyo, Japan) dissolved in 100 mM sodium acetate buffer for 1 h at 37°C prior to Safranin-O staining. Immunohistochemistry was performed on the proximal tibial epiphyseal cartilage of E18.5 embryos using an anti-aggrecan monoclonal antibody (clone 1C6, Developmental Studies Hybridoma Bank at the University of Iowa, USA) and an anti-collagen type X (collagen X) antibody (Sigma-Aldrich). For aggrecan staining, sections were digested with chondroitinase ABC for 1 h at 37°C to unmask the epitope. For collagen X staining, sections were pretreated with 2 mg/ml hyaluronidase (bovine testes, type IV-S; Sigma-Aldrich) at room temperature for 30 min. Subsequently, the sections were treated with 0.3% (v/v) H_2_O_2_ in methanol for 30 min to block endogenous peroxidase. Antigen detection was then carried out by applying aggrecan or collagen X antibodies overnight at 4°C. Detection of these antibodies was performed using the streptavidin-biotin complex method with a Vector M.O.M. Immunodetection Kit (Vector Laboratories, Burlingame, CA, USA). DAB (WAKO) was employed to perform the peroxidase reaction to visualize antigens. Nuclei were counterstained with Mayer’s hematoxylin (WAKO). All sections were observed with a BIOREVO BZ-9000 microscope (KEYENCE).

### Structural analysis of CS chains

Rib cartilage was obtained from E18.5 mice after the administration of general anesthesia, and the cartilage of 4 to 5 mice of each genotype was then mixed. Unsaturated disaccharides in the filtrates were analyzed according to Toyoda’s method [25]. Briefly, freeze-dried rib cartilage was digested with 1 mg/ml Pronase (Calbiochem, CA, USA) for 3 h at 60°C and then filtered through a 0.45-μm membrane. The obtained filtrates were digested with ChaseABC and ChaseAC-II for 2 h at 37°C, and the mixture was then ultrafiltered using a centrifugal ultrafiltration tube. The unsaturated disaccharides derived from CS in rib cartilage were analyzed via HPLC.

### Ki67 and TUNEL staining

For antigen retrieval, sections of the proximal tibial epiphysis were heated in 10 mM citrate buffer (pH 6.0) in an autoclave at 115°C for 10 min. After blocking with 10% normal goat serum/0.1% Tween 20 in PBS for 1 h at room temperature, the sections were stained with an anti-Ki67 antibody (cat. no. NCL-Ki67p, 1:100; Novacastra, UK) overnight at 4°C. Detection was achieved using an Alexa Fluor 488-conjugated secondary antibody (Molecular Probes). The sections were subsequently incubated for 5 min with 0.1% Hoechst 33258 to stain nuclei. The number of Ki67-positive cells was calculated using Dynamic Cell Count BZ-H1C software (KEYENCE). Fluorescent TUNEL assays were performed with a DeadEnd^TM^ Fluorometric TUNEL System (Promega, Fitchburg, WI, USA) according to the manufacturer’s protocol. Images were captured using a BIOREVO BZ-9000 microscope.

### Chondrocyte isolation and primary chondrocyte culture

Chondrocytes from mouse cartilage were isolated and cultured according to a protocol described in a previous report [26]. Briefly, knee cartilage was isolated from E18.5 embryos and digested via two incubations in 3 mg/ml collagenase D (*Clostridium histolyticum*; Roche, Switzerland) in low-glucose Dulbecco’s modified Eagle’s medium (DMEM). Incubation was conducted under a 5% CO_2_ atmosphere in a humidified incubator at 37°C for 45 min. After the cartilage was additionally incubated overnight with 0.5 mg/ml collagenase D/DMEM, the digested chondrocytes were completely separated via pipetting, followed by filtration through a cell strainer with a 0.4-μm pore size (Greiner Bio-One, Austria). Primary chondrocytes were seeded in a 96-well plate, with 8 × 10^3^ cells per well, and cultured in DMEM containing 10% fetal bovine serum. Cell proliferation was assessed using a Cell Counting Kit-8 (Dojindo, Kumamoto, Japan) according to the manufacturer’s instructions, and absorbance values at 450 nm were read with a microplate reader. To quantify GAG production, Alcian blue staining was performed after seven days of culture as described in a previous study [26]. Primary chondrocytes were fixed in Mildform 10 N at room temperature for 15 min, followed by washes with 0.1 N HCl. Sulfated GAG was detected with 1% (w/v) Alcian Blue 8GX in 0.1 N HCl at room temperature for 1 h. After two rinses with 0.1 N HCl, Alcian blue dye was extracted with 6 M guanidine hydrochloride, and absorbance values were quantified at 595 nm using a microplate reader.

### *In situ* hybridization

For the construction of riboprobes for *in situ* hybridization analysis, we prepared two sets of each antisense or sense sequence. The probes were amplified via PCR using the primer sets listed in S4 Table and then subcloned into the pCR^®^4Blunt-TOPO^®^ plasmid vector. Antisense or sense riboprobes were generated using these plasmids as templates in T3 or T7 RNA polymerase-directed *in vitro* transcription mixtures containing a digoxigenin (DIG)-labeled mix (Roche). Hind limbs were fixed in 4% PFA/PBS at 4°C overnight, then maintained in 0.5 M sucrose/PBS at 4°C for cytoprotection and subsequently embedded in O.C.T. compound. Sections with a size of 20 μm treated with 0.5 μg/ml proteinase K (Wako)/0.1% Tween 20 in PBS at 37°C for 10 min and then post-fixed in 4% PFA/PBS for 10 min and rinsed in PBS. Hybridization with DIG-labeled riboprobes was conducted overnight at 55°C in 50% formamide, 5× Denhardt’s solution, 5× SSC, and 50 μg/ml yeast tRNA and then washed with 5× SSC and 2× SSC at 55°C for 15 min each. After washing, the slides were digested with RNase A (20 μg/ml; Sigma-Aldrich) in 0.5 M NaCl/10 mM Tris-HCl pH 7.5/0.1% Tween 20 for 30 min at 37°C. Next, the slides were washed twice with 2× SSC at 42°C for 20 min. After incubation with an alkaline phosphatase-conjugated anti-DIG antibody (Roche), DIG-labeled RNA duplexes were detected with BM purple (Roche).

### Statistical analysis

The results are reported as the mean ± SEM. Statistical analyses were performed using ANOVA or Student’s *t*-test. *P* values are provided in the figure legends and are indicated by asterisks within the figures.

## Results

### *t2* KO mice show normal skeletal development

Previous reports, including those from our group, have shown that *t1* KO mice exhibit slight dwarfism and an approximately 50% decrease in the CS content of cartilage compared with WT mice [6,22]. The gene expression level of *t2*, which is another initiation enzyme *in vitro*, was several times higher than that of *t1* in mouse humeral cartilage at E18.5 (Fig 1B). To further expand this finding, we hypothesized that t2 may have initiation activity *in vivo* as well. To investigate this, we generated *t2* KO mice (Fig 1C), which intriguingly showed normal development, fertility, and growth rates (Fig 1E) compared with WT mice. Analysis of the offspring resulting from crosses among *t2* heterozygotes revealed a Mendelian distribution of WT, heterozygous, and homozygous offspring (S1 Table). In addition, whole body skeletal preparation double-stained with Alizarin red and Alcian blue showed no size differences or skeletal deformities (Fig 1F and 1H).

### *t1*::*t2* double KO mice show severe dwarfism and postnatal lethality

**Fig 2:**
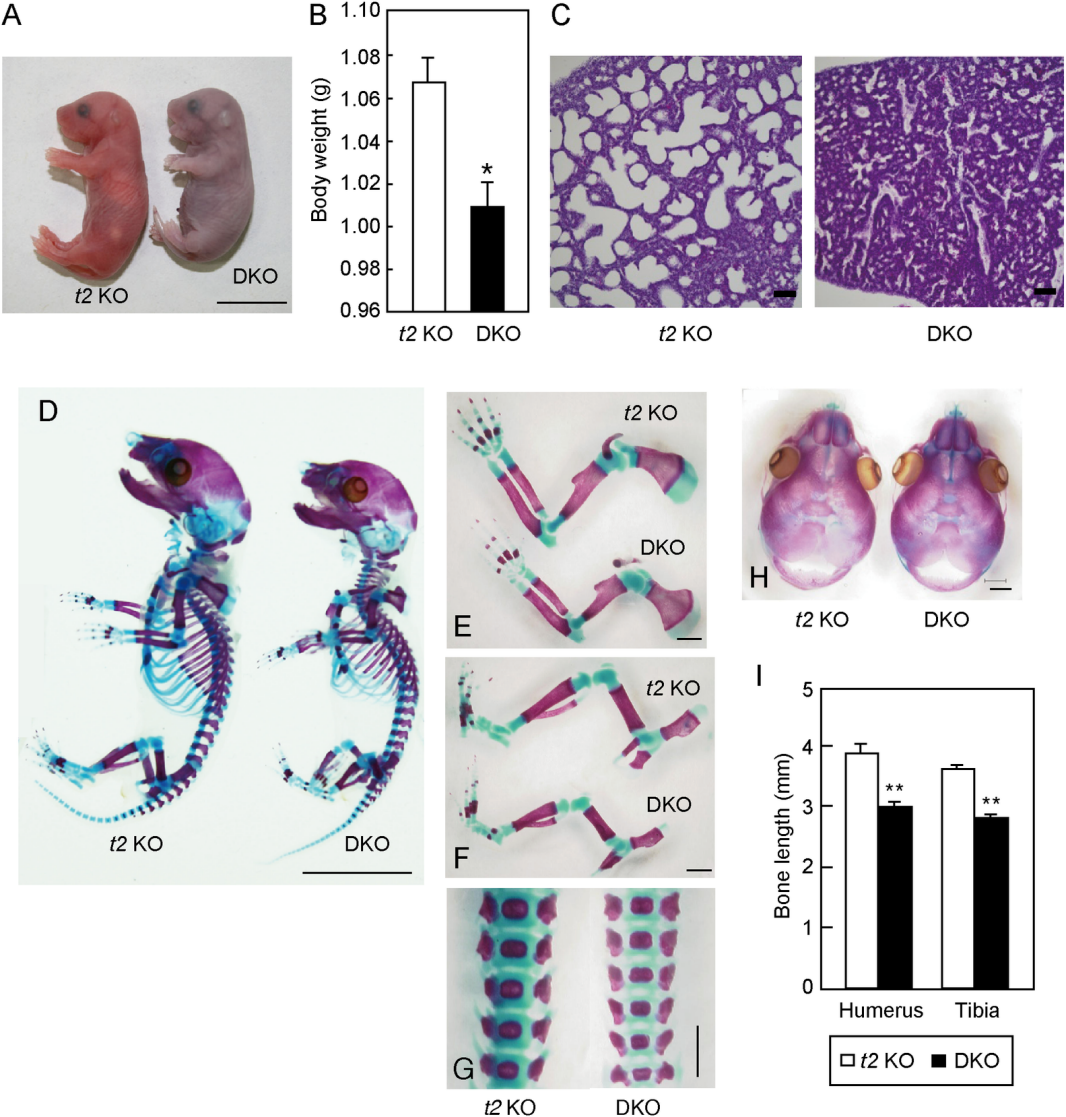
Phenotype of DKO mice. (A) Representative photograph of *t2* KO pups and cyanotic DKO littermates at E18.5. Scale bar: 1 cm. (B) Body weight of *t2* KO (n = 68) and DKO (n = 51) embryos at E18.5. *P < 0.05. (C) Lung sections from *t2* KO embryos and DKO littermates were stained with HE. Scale bar: 100 μm. (D-H) Skeletal whole body (D), upper limb (E), lower limb (F), lumber spine (G), and cranial bone (H) preparation. (I) Humeral and tibial bone length at E18.5 of *t2* KO (n = 5) and DKO (n = 10) mice. **P < 0.01.

If *t2* affects CS initiation activity, *t1*::*t2* double KO mice would be expected to show the same phenotype as *t1* KO mice. Therefore, we generated *t1*::*t2* double KO (DKO) mice. For efficient mating, *t2* KO mice were used as the control group because they show similar skeletal phenotypes to WT mice. Analysis of the offspring resulting from crosses among *t1*^*+/-*^::*t2*^*-/-*^ showed a Mendelian distribution of *t1*^*+/+*^::*t2*^*-/-*^, *t1*^*+/-*^::*t2*^*-/-*^, and *t1*^*-/-*^::*t2*^*-/-*^ offspring during the embryonic period, but *t1*^*-/-*^::*t2*^*-/-*^ mice did not survive after birth (S2 Table). E18.5 embryos were delivered by cesarean section and observed for spontaneous respiration. *t2* KO mice started to breathe spontaneously and showed red skin color after dissection, while DKO mice were cyanosed and died after struggling to initiate breathing (Fig 2A). DKO mice showed slightly but significantly lower body weight than *t2* KO mice (Fig 2B). To investigate the cause of death in DKO mice, their lung sections were stained with hematoxylin and eosin (HE). In *t2* KO lungs, breathing made air flow into alveoli, and a sponge-like appearance was observed (Fig 2C). Conversely, DKO mice showed no alveolar air spaces in their lungs, indicating that DKO mice died of respiratory failure. Although skeletal preparation showed no skeletal deformity, the whole body size of DKO mice was slightly smaller than that of *t2* KO mice (Fig 2D and 2H). The humeral and tibial lengths of DKO mice were significantly reduced by 22% compared with those of *t2* KO mice (humerus; *t2* KO: 3.93 ± 0.04 mm, n = 5, DKO: 3.07 ± 0.02 mm, n = 10, tibia; *t2* KO: 3.64 ± 0.04 mm, n = 5, DKO: 2.81 ± 0.02 mm, n = 10) (Fig 2I). Previous data indicated there was no difference in bone length between WT and *t1* KO embryos. Therefore, our DKO embryo showed a severe phenotype compared with *t1* KO embryos, signifying the necessity of t2 in skeletal development processes.

### Chondrocyte-specific *t1*::*t2* double KO mice show phenotypes similar to DKO mice, with a number of surviving progeny

**Fig 3:**
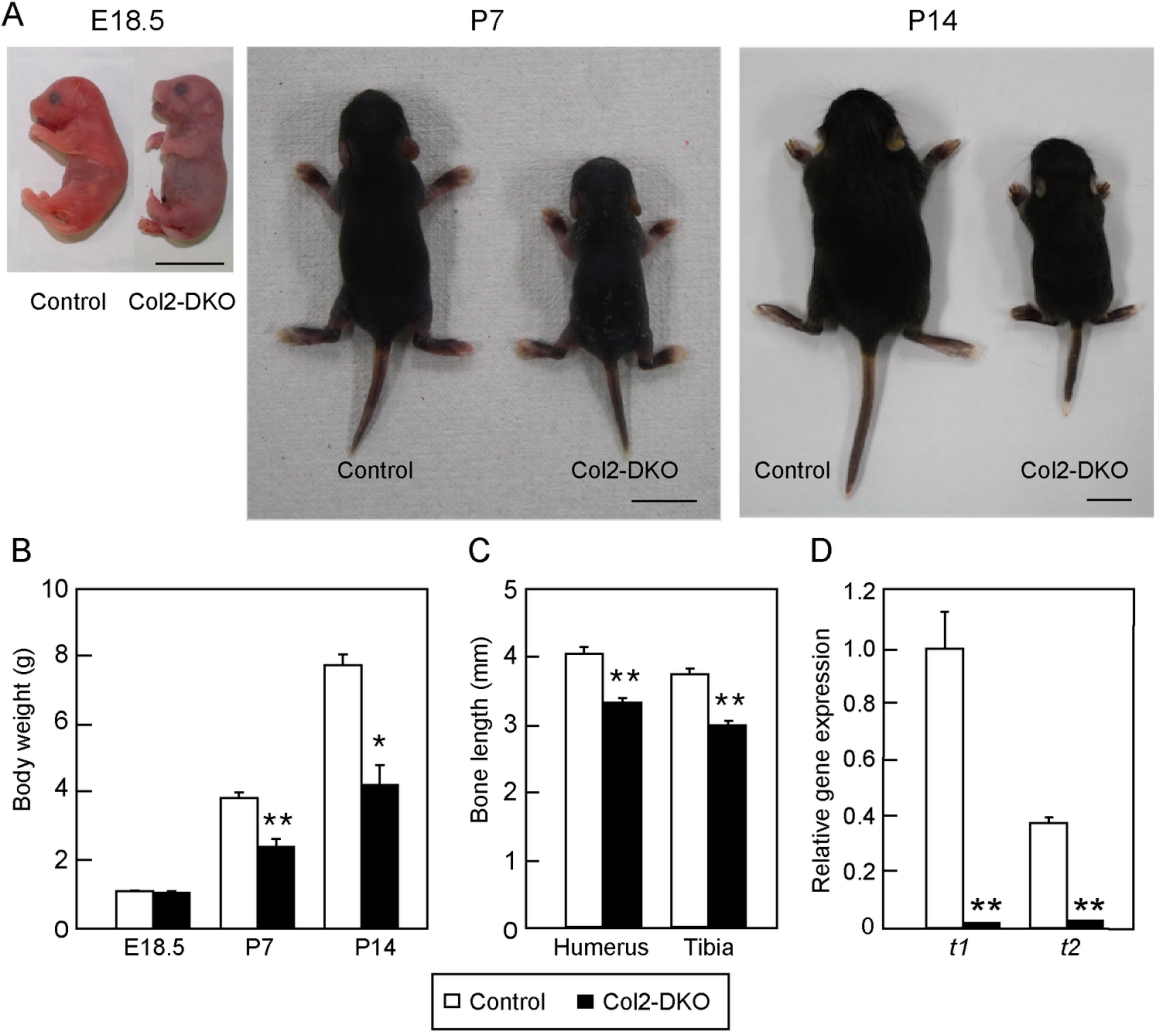
Phenotype of Col2-DKO mice. (A) Representative photograph of Control pups and Col2-DKO littermates at E18.5, P7, and P14. Scale bar: 1 cm. (B) Body weight of Control (E18.5; n = 16, P7; n = 4, P14; n = 4) and Col2-DKO (E18.5; n = 12, P7; n = 4, P14; n = 4) mice at various stages. *P < 0.05, **P < 0.01. (C) Humeral and tibial bone length at E18.5 of Control (n = 6) and Col2-DKO (n = 7) mice. (D) Quantitative analysis of *t1* and *t2* transcripts in the knee cartilage of WT (n = 3) and Col2-DKO (n = 3) mice at E18.5 using real-time RT-PCR. The expression of each transcript was normalized to that of β-actin. **P < 0.01.

Normal cartilage development is indispensable for bone elongation, which was abnormal in DKO mice. CS is widely known to be expressed in cartilage and *t1* KO and *Css1* KO mice were reported to exhibit a deformed skeletal system as a result of abnormal cartilage development [6,22,27]. To investigate the cartilage-specific function of t1 and t2, we generated chondrocyte-specific *t1*::*t2* double KO mice (Col2-DKO) using *Collagen2a1*-Cre mice [28]. Since heterozygous KO of *t1* or *t2* showed the same phenotype, WT, *t1*^*+/-*^::*t2*^*+/+*^, *t1*^*+/+*^::*t2*^*+/-*^, and *t1*^*+/-*^::*t2*^*+/-*^ mice were used as control groups (Control). E18.5 embryos of Control mice started spontaneous respiration after cesarean section (Fig 3A). Forty-three Col2-DKO mice among 53 immediately died of respiratory failure after birth. However, the remaining 10 Col2-DKO mice started spontaneous respiration. Col2-DKO mice rarely survived to adulthood and died by P14 in most cases. Although Col2-DKO mice showed the same body weight as Control mice at E18.5, they showed significantly (50%) lower body weight than Control mice and severe dwarfism at P7 (Control: 3.80 ± 0.15 g, n = 4, Col2-DKO: 2.40 ± 0.22 g, n = 4) and P14 (Control: 7.70 ± 0.30 g, n = 4, Col2-DKO: 3.80 ± 0.15 g, n = 4) (Fig 3B). At E18.5, when body weight showed no difference between Col2-DKO and Control mice, skeletal preparation of humeri and tibiae from Col2-DKO mice were shorter than those of Control mice (humerus; Control: 4.06 ± 0.09 mm, n = 6, Col2-DKO: 3.28 ± 0.08 mm, n = 7, tibia; Control: 3.72 ± 0.09 mm, n = 6, Col2-DKO: 2.97 ± 0.08 mm, n = 7) (Fig 3C). The quantitative RT-PCR analysis of knee cartilage showed no or slight *t1* and *t2* gene expression in Col2-DKO mice compared with those of WT mice, and thus, chondrocyte-specific *t1*::*t2* KO was confirmed (Fig 3D). These results indicate that deficiency of both t1 and t2 in chondrocytes not only affects endochondral ossification during the fetal period but also influences bone growth after birth.

### Impaired CS content in cartilage and induction of abnormal endochondral ossification by *t1*::*t2* double deficiency

**Fig 4:**
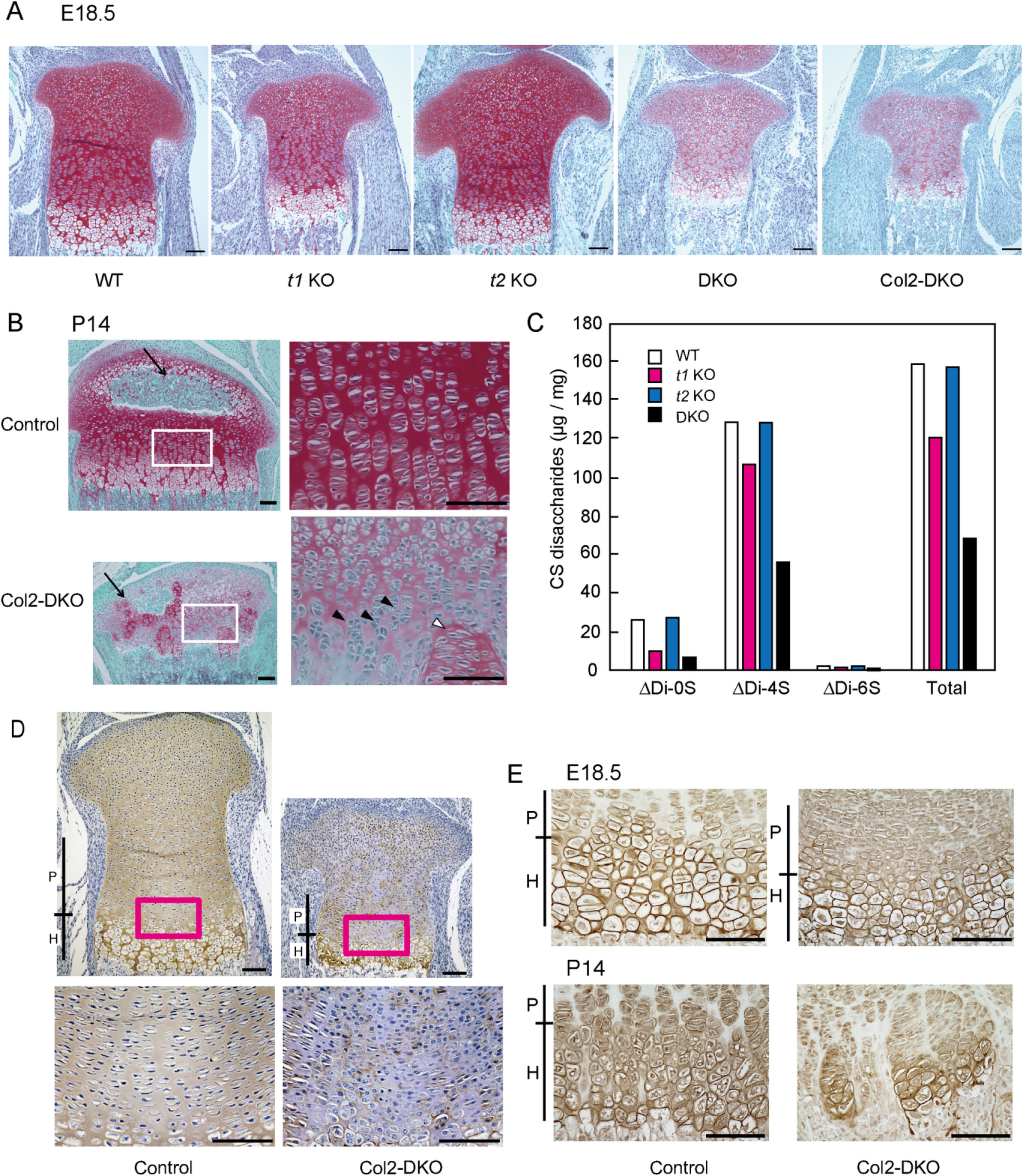
Safranin-O staining, CS content, and immunohistochemical analyses in cartilage. (A) Safranin-O staining in E18.5 proximal tibial cartilage of WT, *t1* KO, *t2* KO, DKO, and Col2-DKO mice. (B) Safranin-O staining in P14 tibial cartilage of Control and Col2-DKO mice. Arrows indicate secondary ossification centers. Each right panel is a higher magnification image of the regions labeled with white squares in the left panel. Black arrowheads indicate abnormal shape and cell layer structure of the growth plate in Col2-DKO mice. White arrowheads indicate ectopic localization of proliferating chondrocytes in the hypertrophic zone. (C) The total amount and disaccharide analysis of the rib cartilage in WT, *t1* KO, *t2* KO and DKO mice. Δdi-0S, ΔHexAα1-3GalNAc; Δdi-4S, ΔHexAα1-3GalNAc(4S); Δdi-6S, ΔHexAα1-3GalNAc(6S). (D) Immunohistochemical aggrecan staining using 1C6 monoclonal antibody. The sections from Control and Col2-DKO mice were used for staining after chondroitinase ABC treatment. Each lower panel shows a higher magnification image of the regions labeled by magenta squares in the upper panel. Scale bar: 100 μm. (E) Immunostaining with an antibody against type X collagen in Control and Col2-DKO cartilage at E18.5 and P14. P, Proliferative chondrocyte; H, Hypertrophic chondrocyte. Scale bar: 100 μm.

Since t1 and t2 function in cartilage is crucial for skeletal development, we compared CS content in each genotype of mice including WT, *t1* KO, *t2* KO, and DKO at E18.5 by performing histological analysis of the proximal tibial growth plate with Safranin-O staining. Safranin-O is a red stain that detects the GAG content of cartilage ECM. To verify the GAG that we are detecting, we treated the samples with chondroitinase ABC, a CS- and hyaluronic acid (HA)-specific digestive enzyme, because it is possible that Safranin-O staining detects GAGs other than CS, such as heparan sulfate. Safranin-O staining of chondroitinase ABC-treated samples diminished, indicating that the Safranin-O staining in this condition was certainly CS and HA derived from cartilage ECM (S1 Fig). Once the specificity of the Safranin-O staining was confirmed in WT mice, other genotypes were also stained with Safranin-O, and the staining intensity was similar in both *t2* KO and WT mice (Fig 4A). For the case of t*1* KO mice, a slight decrease in intensity compared with that in WT mice was observed. The staining intensity revealed that the largest difference when comparing DKO mice to WT mice was a weaker intensity compared with *t1* KO mice. Structural organization of the proliferative and hypertrophic zones of the DKO mice did not show any significant changes compared with WT mice; however, the size of their epiphysis was smaller than that of WT mice (Fig 4B). A drastic decrease in Safranin-O staining was also noticed in Col2-DKO mice, which exhibit phenotypes similar to those of the DKO mice mentioned above. These results indicate that the existence of GAG is correlated with the skeletal phenotype, where a lower amount of CS leads to a more severe defect in skeletal development.

To investigate postnatal endochondral ossification of Col2-DKO cartilage, where Safranin-O staining intensity was the lowest at E18.5, Safranin-O staining of Col2-DKO tibial growth plates at P14 was performed. In addition to the smaller epiphyseal size, decreased staining intensity and stained area was observed in Col2-DKO mice compared with those of Control mice (Fig 4B). The formation of secondary ossification centers was also delayed in Col2-DKO mice (Fig 4B, arrow). Moreover, Col2-DKO growth plates showed drastically disrupted proliferative and hypertrophic zones. The nucleus and cytoplasm of proliferating chondrocytes were flattened in shape and arranged into vertical columns in Control growth plates, while those of Col2-DKO mice showed round cell shapes and an absence of longitudinal orientation (Fig 4B, black arrowhead). Some chondrocytes exhibited a flat cell shape, and the ECM surrounding these cells displayed strong Safranin-O staining, but the cells were misplaced in the region outside of the proliferative zone (Fig 4B, white arrowhead). To clarify the CS synthesis ability in t1 and t2 *in vivo*, the disaccharide composition of CS was determined using rib cartilage extracts from WT, *t1* KO, *t2* KO and DKO mice. The total CS amount per dry weight of *t1* KO, *t2* KO and DKO mice was 74.7%, 99.6% and 40.3% that of WT, respectively (Fig 4C). The relative amount of C0S, C4S, and C6S in each mouse was as follows: 16.9%, 81.3%, and 1.8% for WT; 8.0%, 90.2%, and 1.8% for *t1* KO; 17.1%, 81.3%, and 1.7% for *t2* KO; and 10.7%, 80.7%, and 1.6% for DKO (Fig 4C). These results indicate that the CS amount in DKO cartilage (40.3% of WT) was further reduced than the decrease observed in *t*1 KO cartilage (74.7% of WT), although the disaccharide composition of *t1* KO, *t2* KO and DKO cartilage was similar to that of WT. To determine how CS reduction causes the abnormal endochondral ossification described above, immunohistochemical staining of aggrecan, a core protein of CS and the main component of cartilage ECM, was conducted on tibial growth plates at E18.5. WT mice showed uniform aggrecan expression in cartilage ECM in growth plates. In contrast, most portions of the Col2-DKO ECM were revealed to be aggrecan-negative, and scarce aggrecan-positive regions were restricted to the periphery of the cartilage lacunae (Fig 4D). These findings indicate that CS loss induced by *t1* and *t2* KO disturbs aggrecan core protein distribution throughout the cartilage ECM. To assess the differentiation stages of chondrocytes, we next performed immunohistochemical staining of collagen X, a marker for hypertrophic chondrocytes, using tibial growth plates at E18.5 and P14. No difference was observed between Col2-DKO and WT mice at E18.5. At P14, collagen X expression was distributed irregularly in Col2-DKO mice, whereas a uniformly stained hypertrophic zone was seen in WT mice (Fig 4E). These results suggest that both t1 and t2 function in chondrocytes are required for CS synthesis *in vivo*, and their loss induces abnormal endochondral ossification, including disrupted growth plate structure and differentiation stages.

### Cartilage-specific *t1*::*t2* double deficiency induces reduced proliferation and increased apoptosis of chondrocytes

**Fig 5:**
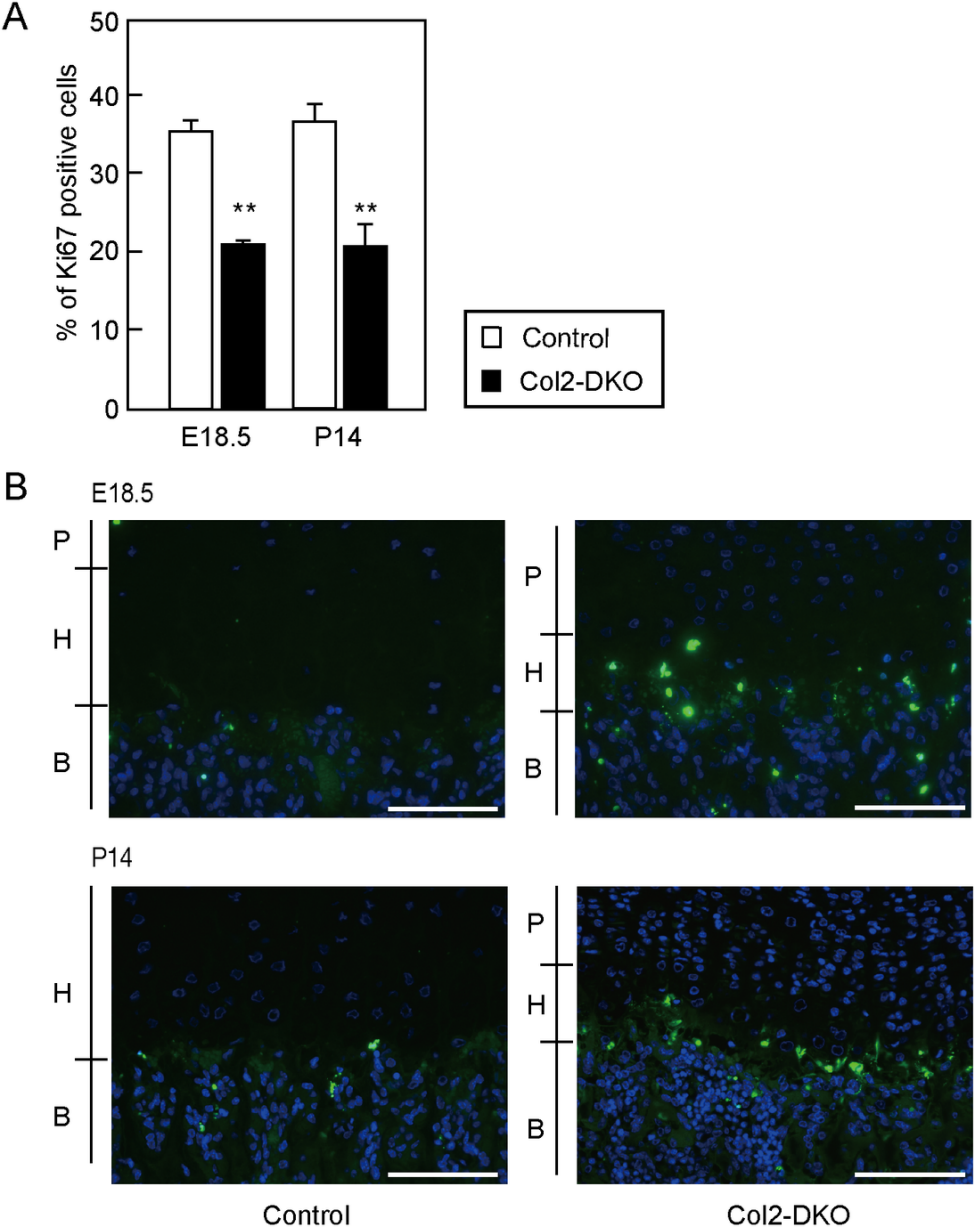
Cell proliferation and apoptosis in proximal tibial epiphyseal cartilage. (A) Quantification of cell proliferation assessed by Ki67 immunostaining in tibia sections from Control (E18.5; n = 4, P14; n = 3) and Col2-DKO (E18.5; n = 3, P14; n = 6) mice at P14. **P < 0.01. (B) TUNEL staining of tibia sections from Control (n = 3) and Col2-DKO (n = 3) mice at E18.5 and P14. P, Proliferative chondrocyte; H, Hypertrophic chondrocyte; B, Bone. Scale bar: 100 μm.

Abnormal endochondral ossification and reduced epiphyseal size, which were found in Col2-DKO mice, often reflect alterations in the balance of proliferation versus apoptosis. To test this, we performed immunohistochemical staining of Ki67, a marker of proliferating cells, and terminal dUTP nick-end labeling (TUNEL) staining using tibial growth plates at E18.5 and P14. Col2-DKO mice displayed an approximately 50% reduction in the percentage of Ki67-positive cells in the proliferative zone compared with WT mice at both E18.5 (Control: 35.4 ± 1.3%, n = 4, Col2-DKO: 19.7 ± 1.6%, n = 3, *P* < 0.001) and P14 (Control: 36.6 ± 2.5%, n = 3, Col2-DKO: 21.1 ± 2.7%, n = 6, *P* < 0.001) (Fig 5A). When apoptosis was examined with TUNEL staining, Col2-DKO mice displayed increased apoptosis in the hypertrophic zone at both E18.5 and P14, while Control mice exhibited moderate apoptosis (Fig 5B). These results indicate that the CS reduction caused by *t1* and *t2* KO leads to decreased proliferation and accelerated apoptosis in chondrocytes, contributing to the disrupted endochondral ossification and morphology of the growth plate.

### Characterization of primary chondrocyte culture from Col2-DKO mice

**Fig 6:**
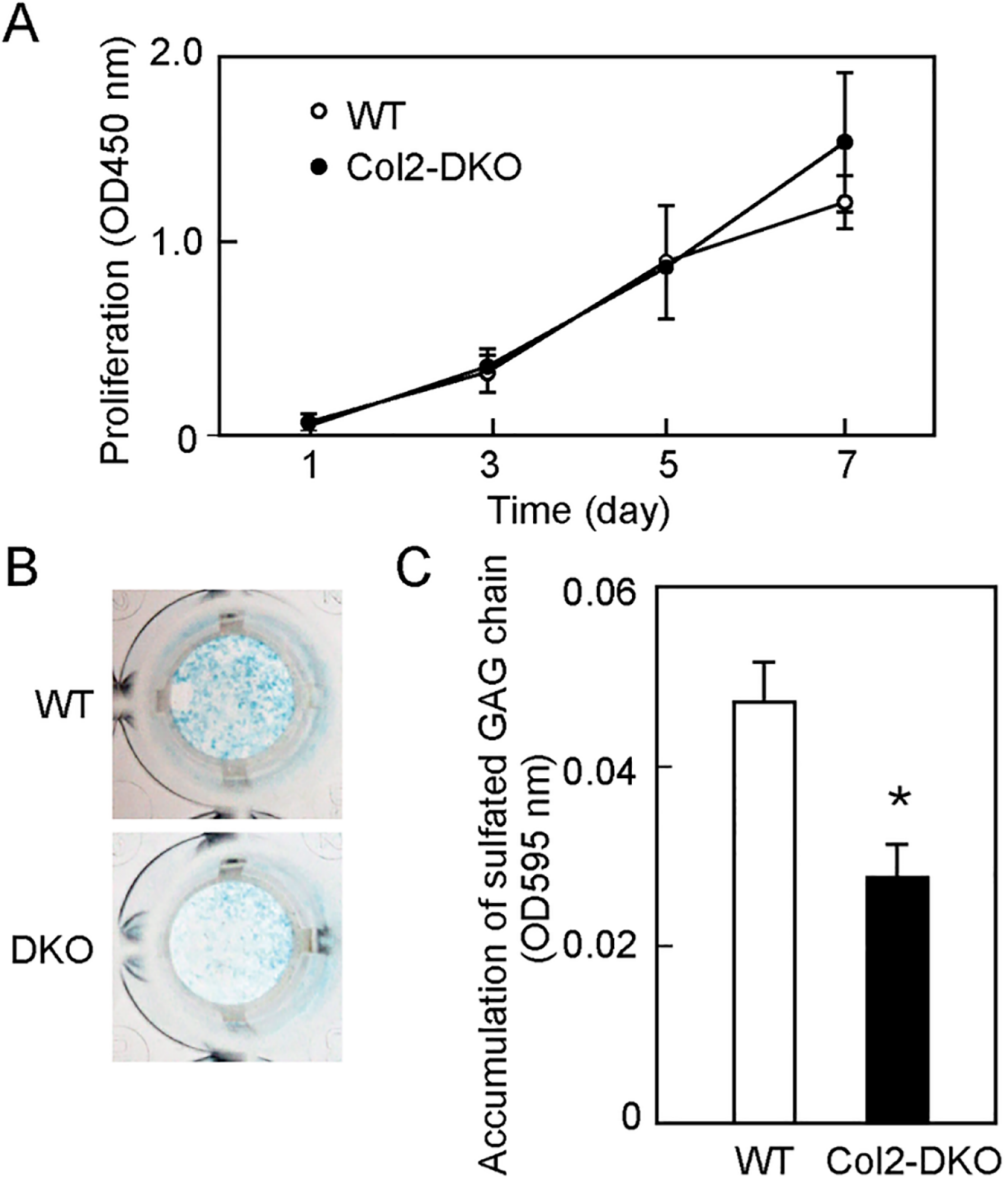
GAG accumulation and cell proliferation in primary chondrocyte cultures. (A) Proliferation of WT (n = 3) and Col2-DKO (n = 3) chondrocytes. (B) Primary chondrocytes from knee cartilage of WT and Col2-DKO mice at E18.5 were stained for GAG accumulation using Alcian blue at culture day 7. (C) The absorption intensity at 595 nm of Alcian blue-stained cell extracts in WT (n = 3) and Col2-DKO (n = 3) mice was measured to quantify sulfated GAG accumulation. *P < 0.05.

The above findings further led us to the hypothesis that disrupted aggrecan causes a disordered ECM environment, resulting in abnormal endochondral ossification in Col2-DKO mice. To test this, primary chondrocytes from knee and femur cartilage at E18.5 were cultured, and their ability to respond to exogenous factors was examined. The accumulation of sulfated GAG in primary chondrocyte cultures was quantified through Alcian blue staining, and the amount of sulfated GAG was found to show a significant decrease of approximately 60% in Col2-DKO primary chondrocyte cultures compared with WT mouse chondrocytes (WT: 0.047 ± 0.004, n = 3, Col2-DKO: 0.028 ± 0.004, n = 3) (Fig 6A and 6B). Growth rate measurements of these chondrocyte cultures revealed no difference between Col2-DKO and WT cells (Fig 6C). These results show that although the CS amount is reduced in Col2-DKO mice, their chondrocytes can respond to extracellular cues.

### Quantitative RT-PCR and *in situ* hybridization analyses of humeral cartilage

**Fig 7:**
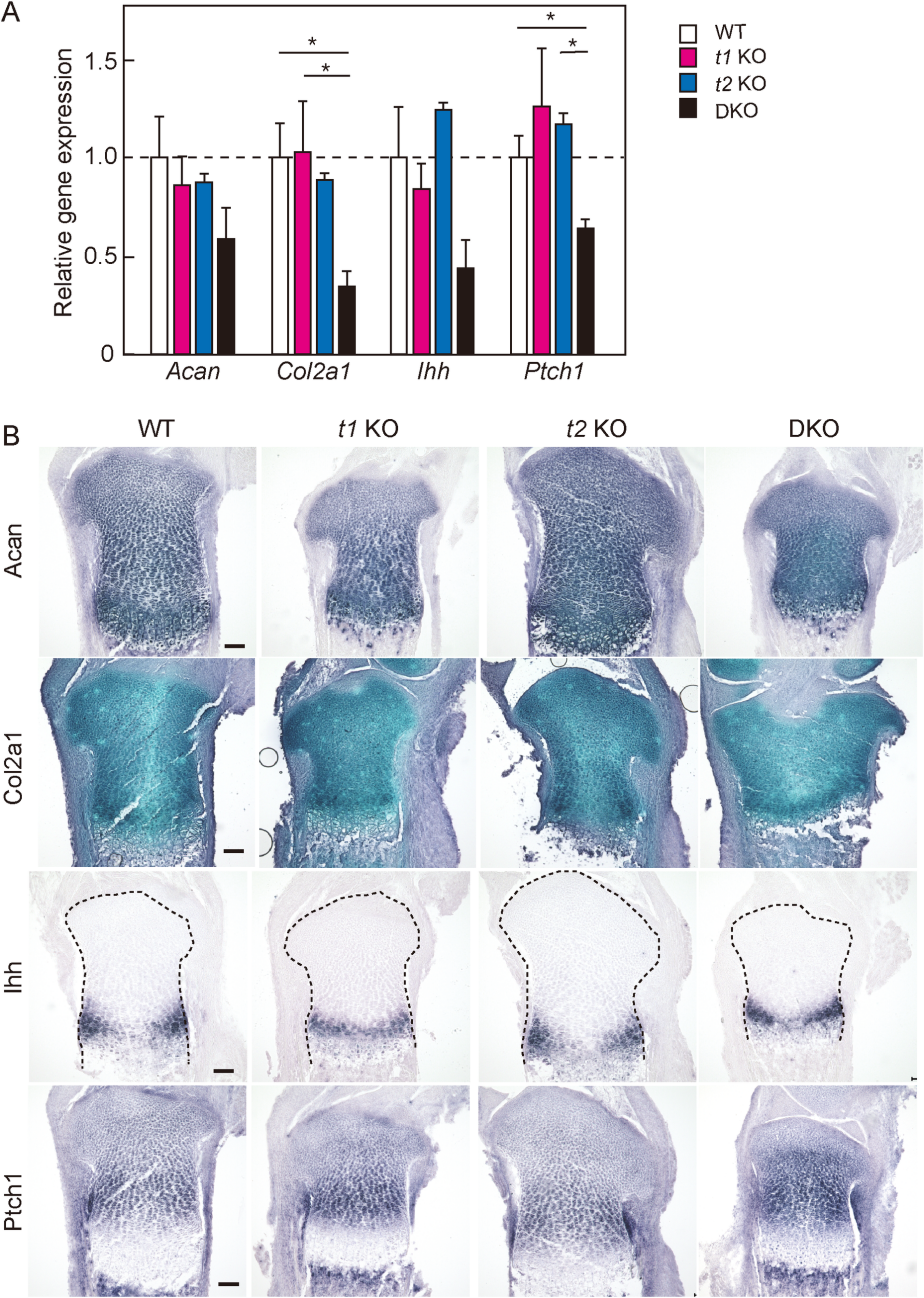
Quantitative gene expression and *in situ* hybridization of genes associated with endochondral ossification. (A) Quantitative analysis of transcription of ECM and Ihh signaling molecules in E18.5 humeral cartilage using real-time RT-PCR. The expression of each transcript was normalized to that of *Hprt*. The amount of each transcript in WT cartilage was set to a value of 1.0. *P < 0.05. (B) The expression of the indicated probes was examined by *in situ* hybridization in tibial sections at E18.5. The black dotted lines indicate the contour of the proximal epiphysis of the tibia. Scale bar: 100 μm.

To determine which process was affected in the abnormal endochondral ossification observed in DKO mice, the transcriptional levels of major ECM molecules in cartilage were investigated in humeral cartilage using quantitative real-time PCR. The expression of aggrecan (*Acan*) and collagen type II α1 chain (*Col2a1*) in *t1* KO and *t2* KO mice was found to be similar to that in WT mice (Fig 7A), but the expression of *Col2a1* was prone to decrease in DKO cartilage compared with WT cartilage (*Acan* (F^[^^3^^,^ ^23^^]^ = 0.75, *P* = 0.53), *Col2a1* (F^[^^3^^,^ ^23^^]^ = 2.79, *P* = 0.06)). However, *in situ* hybridization showed that the distribution of *Acan* and *Col2a1* transcripts in *t1* KO, *t2* KO or DKO tibial cartilage was also similar to that in WT cartilage (Fig 7B). The transcripts of Indian hedgehog (*Ihh*) and one of the hedgehog target genes, *Ptch1*, were also expressed at low levels in DKO cartilage compared with WT cartilage, but these differences were not statistically significant (*Ihh* (F^[^^3^^,^ ^23^^]^ = 1.38, *P* = 0.27), *Ptch1* (F^[^^3^^,^ ^23^^]^ = 2.75, *P* = 0.07)). Moreover, the distribution of *Ihh* and *Ptch1* transcripts was normal in DKO cartilage. When comparisons between WT and DKO were performed using Student’s *t*-test, the expression of *Col2a1* and *Ptch1* was found to be significantly decreased in DKO mice. To clarify whether other CS synthases can compensate for a loss in *t1*, *t2* or both genes, the expression of six CS synthase transcripts in WT, *t1* KO, *t2* KO, and DKO humeral cartilage at E18.5 was measured using real-time RT-PCR (S2 Fig). While complete loss was observed in *t1* expression in *t1* KO and DKO or *t2* expression in *t2* KO and DKO, *t1* expression in *t2* KO or *t2* expression in *t1* KO did not change compared with that in WT. No change was observed *Css1*, *Css2*, *Css3* and *Csglcat* gene expression between each mouse genotype.

## Discussion

In this study, we generated and analyzed mice lacking *t2* or both *t1*::*t2* gene expression to elucidate the role of CS in cartilage. Previously, *t1* KO mice were found to exhibit slight dwarfism due to minor impairment of endochondral ossification and reduction of cartilaginous CS content [6]. According to an *in vitro* enzymatic characterization, among the six CS glycosyltransferases, only t1 and t2 possessed the ability to independently initiate CS synthesis. We previously reported that t1 efficiently transfers GalNAc onto the linkage tetrasaccharide *in vitro*, which is common to both CS and heparin sulfate/heparin, and t1 initiation activity is stronger than that of t2 [15,17]. *t1* KO mice demonstrated a similar or slightly increased CS chain length, although the CS levels of CS in *t1* KO cartilage decreased to ~50% those in normal mouse cartilage [6]. The remaining CS contents in *t1* KO cartilage are predicted to be synthesized by other CS glycosyltransferases. t2 is most likely to possess initiation activity for CS synthesis *in vivo* and predicted to affect endochondral ossification as well as t1. However, *t2* KO mice showed no apparent structural cartilage abnormality. Although Izumikawa *et al*. [29] demonstrated the proportion of linkage region saccharide in *t1* KO cartilage was changed compared with that of WT, it was not changed between WT and *t2* KO mice. These results suggest that t1 has higher specific activity toward initiation of CS synthesis than t2 *in vivo*. Therefore, the CS synthesis ability of t2 might almost be compensated for by t1, but t1 has sufficient CS synthetic ability even if t2 is absent. Takeuchi et al. reported that their *t1* KO exhibited reduced scar formation after spinal cord compression injury [30]. They also generated *t2* KO mice, which showed normal CS production in areas with glial scars. Their study described a spinal cord-specific *t2* KO phenotype but did not mention cartilage development. These data suggest that t2 does not individually influence CS biosynthesis in cartilage *in vivo*. On the other hand, t2 has elongation activity and regulates CS chain length [31]. However, our data showed that although the CS content was exactly the same between WT and *t2* KO cartilage, the CS content in DKO cartilage was less than that in *t1* KO cartilage. These data suggest that a synergistic effect on CS synthesis might be caused by coordination of t1 and t2. In WT cartilage, there might be another initiation enzyme to generate a heterodimer with t1 preferentially. This other initiation enzyme may function by combining with t2 when t1 is lost, but its CS synthesis ability is lower than that of the t1 heterodimer. If the other initiation enzyme fails to form a complex with t1 or t2, initiation activity might be lost.

DKO mice exhibited smaller body size than *t1* KO mice and postnatal lethality. The Safranin-O staining intensities and CS content in DKO cartilage were lower than those in *t1* KO cartilage, whereas those in *t2* KO cartilage were similar to the levels in WT cartilage. *Slc35d1* null mice, which encode an endoplasmic reticulum nucleotide-sugar transporter that transports UDP-GlcA and UDP-GalNAc, show a drastic decrease in CS content, extremely short limbs, and no survival in the neonatal period [32]. The skeletal formation of *Slc35d1* KO mice showed a more severe defect than our DKO mice, suggesting that the CS content in DKO cartilage was not completely lacking. These observations indicate that residual CS content in DKO cartilage is synthesized by other CS glycosyltransferases as initiation enzymes. There are only two CS glycosyltransferases in *C*. *elegans*, cChSy and PAR2.4, which are orthologous to CSS1 and CSS2, respectively. *cChSy*-RNAi and *PAR2*.*4*-RNAi worms showed decreases in chondroitin of 73% and 54%, respectively [33], indicating that CSS1 or CSS2 might be essential for CS biosynthesis. The heterodimers among four enzymes, CSS1, CSS3, CSGlcAT and CSS2, synergistically increased CS elongation activity [34]. Moreover, the heterodimer of the CSGlcAT/CSS2 complex has initiation activity *in vitro* [19]. However, the formation of a t1 and t2 heterodimeric complex for hyperactivity of CS initiation *in vitro* has not been reported.

Some researchers have reported the phenotype of cartilage in CS glycosyltransferase null mice. *Css1* KO mice display multiple skeletal defects including chondrodysplasia and decreased bone density due to an imbalance in chondroitin sulfation [27]. However, *Css2* KO mice exhibited no overt abnormalities, while the CS content was slightly decreased in cartilage to 80% that of WT mice [21]. Biochemical analysis of *Css2* KO primary chondrocyte cultures showed that *Css1* regulates both initiation and elongation activity.

All DKO and almost all Col2-DKO mice died just after birth due to respiratory failure, and they exhibited severe dwarfism due to insufficient endochondral ossification. Degradation of aggrecan core protein was observed in *t1* KO cartilage [6], suggesting that aggrecan metabolism was further changed in DKO cartilage. *Cartilage matrix deficiency* (*cmd*/*cmd*) mice, with a null mutation of the aggrecan gene, exhibited perinatal lethal dwarfism and craniofacial abnormalities [35,36]. Recently, Lauing et al. reported that the cause of lethality was severe tracheomalacia and tracheal stenosis [37]. However, DKO and Col2-DKO mice exhibited slight tracheal obstruction but normal tracheal development (S3 Fig). The brachymorphic mouse, with spontaneous mutation in phosphoadenosine phosphosulfate (PAPS) synthase 2, which synthesizes the sulfate donor PAPS for GAG sulfation, showed postnatal chondrodysplasia, reduction in sulfation level on CS in cartilage, and abnormal Indian hedgehog (Ihh) distribution in cartilage ECM [38]. Moreover, recombinant Ihh protein was bound to aggrecan via its CS chains, indicating that Ihh signaling plays an important role in growth plate development. We observed that *Acan*, *Col2a1*, *Ihh*, and *Ptch1* transcripts were prone to be downregulated in DKO cartilage. Our results suggest that the abnormal endochondral ossification observed in DKO mice may also play a role in the reduction of Ihh signaling for cartilage development, in addition to the decreased expression of ECM molecules.

CS and HS synthesis utilizes an identical tetrasaccharide linkage region, and each initiation enzyme competes for accepter substrate. HS production was increased in injured spinal cord *t1* KO mice, replacing CS reduction [30]. HS synthases are also expressed in cartilage, and HS is indispensable for normal limb formation [39]. Our Safranin-O staining after chondroitinase ABC treatment showed no increase in staining signals in Col2-DKO cartilage at E18.5 compared with that in WT, indicating that GAG on CSPGs in DKO cartilage is not replaced by HS chains to substitute for CS chains. This result suggests that either CS or HS transfer to the linkage region is not due to competition of both initiation enzymes in cartilage.

At P14, columnar proliferating chondrocytes were scattered in Col2-cre cartilage, and the ECM around it was strongly stained with Safranin-O. The Safranin-O staining intensity was similar to that of ECM in WT proliferating chondrocytes. The cause of aberrant GAG expression is thought to be due to mosaic ablation of t1 and t2 in chondrocytes by Col2-cre recombinase driver mice.

We observed that chondrocyte proliferation was reduced in Col2-DKO mice, as demonstrated by the reduction in Ki67-positive chondrocytes. Because cell apoptosis is increased in the hypertrophic zone of Col2-DKO mice, the reduced epiphyseal size of Col2-DKO mice might be due to the combined effect of a decreased number of chondrocytes entering the hypertrophic zone and increased chondrocyte apoptosis.

We have shown for the first time that CS synthases other than t1 and t2 may exhibit initiation activity *in vivo*. There are inherited diseases in humans associated with mutations in the CSGALNACT1 gene [40], and a decrease in chondroitin sulfate is associated with osteoarthritis in humans [41]. *In vitro* experiments have demonstrated that overexpression of the *t1* gene enhances chondroitin sulfate synthesis [20]; thus, identification of the combination of enzymes with the best ability to synthesize chondroitin sulfate may make it possible to induce chondroitin sulfate synthesis *in vivo*. The application of these findings is expected to lead to the development of therapeutic and preventive methods for these diseases. Therefore, the demonstration of the CS synthetic abilities of t1 and t2 *in vivo* is important for complete elucidation of the mechanism of chondroitin sulfate biosynthesis.

## Supporting information

S1 Fig Quantitative analysis of the expression of six CS synthases in E18.5 humeral cartilage. The amount of each transcript in WT cartilage was set to a value of 1.0. WT (n = 6), *t1* KO (n = 5), *t2* KO (n = 7), DKO (n = 9). ND; Not detected.
(TIF)

S2 Fig Safranin-O staining of WT tibial cartilage at E18.5 without and with chondroitinase ABC pretreatment. (TIF)

S3 Fig Safranin-O staining of tracheal cartilage at E18.5. (TIF)

S1 Table Genotypic analysis of offspring from *Csgalnact-1* or *Csgalnact*-2 heterozygous intercrosses. (XLSX)

S2 Table *Csgalnact-1*::*Csgalnact-2* double-deficient embryos die at birth. (XLSX)

S3 Table Primer information for real-time PCR. (XLSX)

S4 Table Primer sequences for *in situ* hybridization. (XLSX)
