# Supplementary figures and images for: Postnatal lethality and chondrodysplasia in mice lacking both chondroitin sulfate N-acetylgalactosaminyltransferase-1 and -2
Source: PLoS One. 2017 Dec 29;12(12):e0190333. doi: 10.1371/journal.pone.0190333 (PMC5747463; doi:10.1371/journal.pone.0190333)

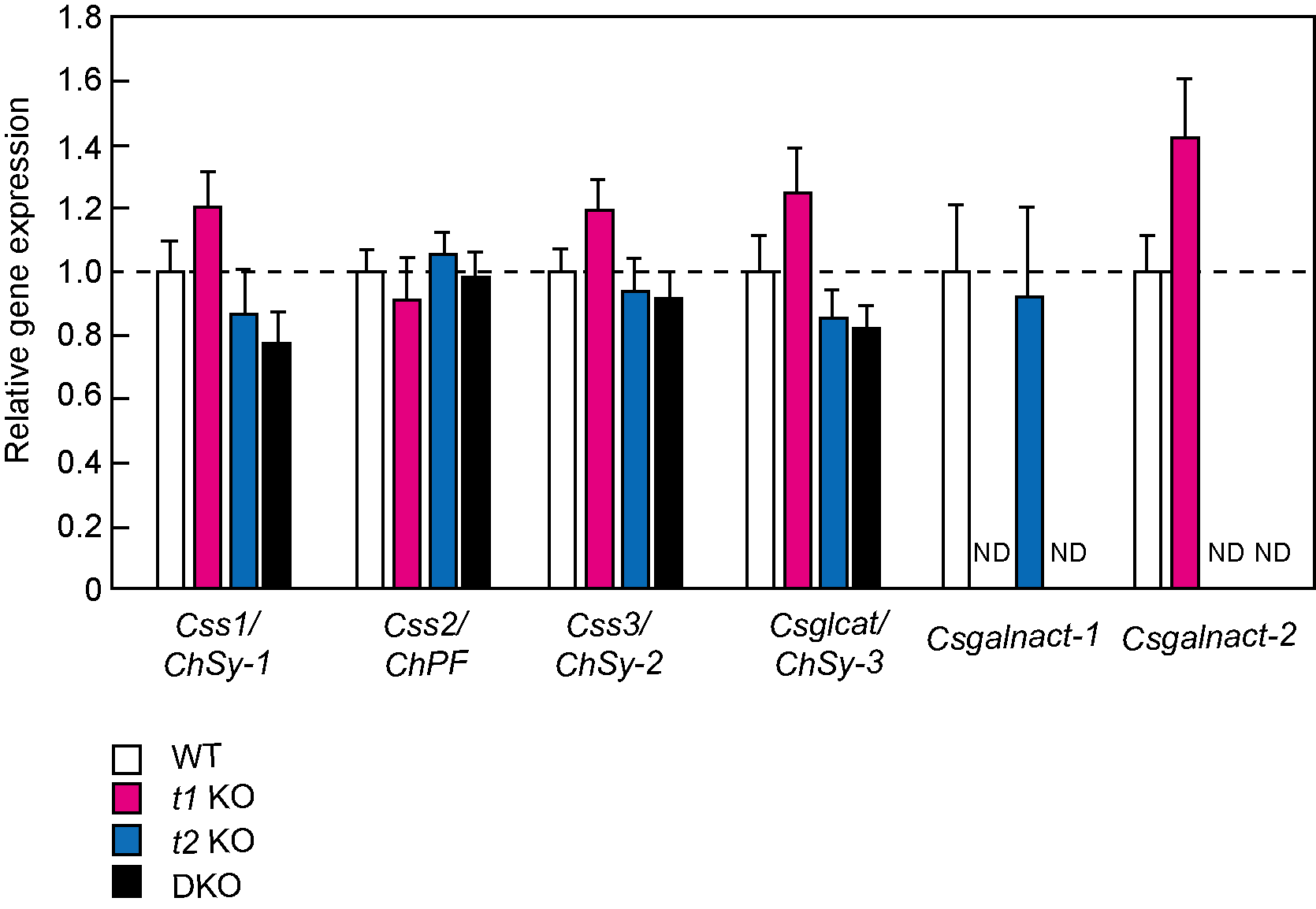

Supplement: S1 Fig — The amount of each transcript in WT cartilage was set to a value of 1.0. WT (n = 6), t1 KO (n = 5), t2 KO (n = 7), DKO (n = 9). ND; Not detected. (TIF) [file pone.0190333.s001.tif]

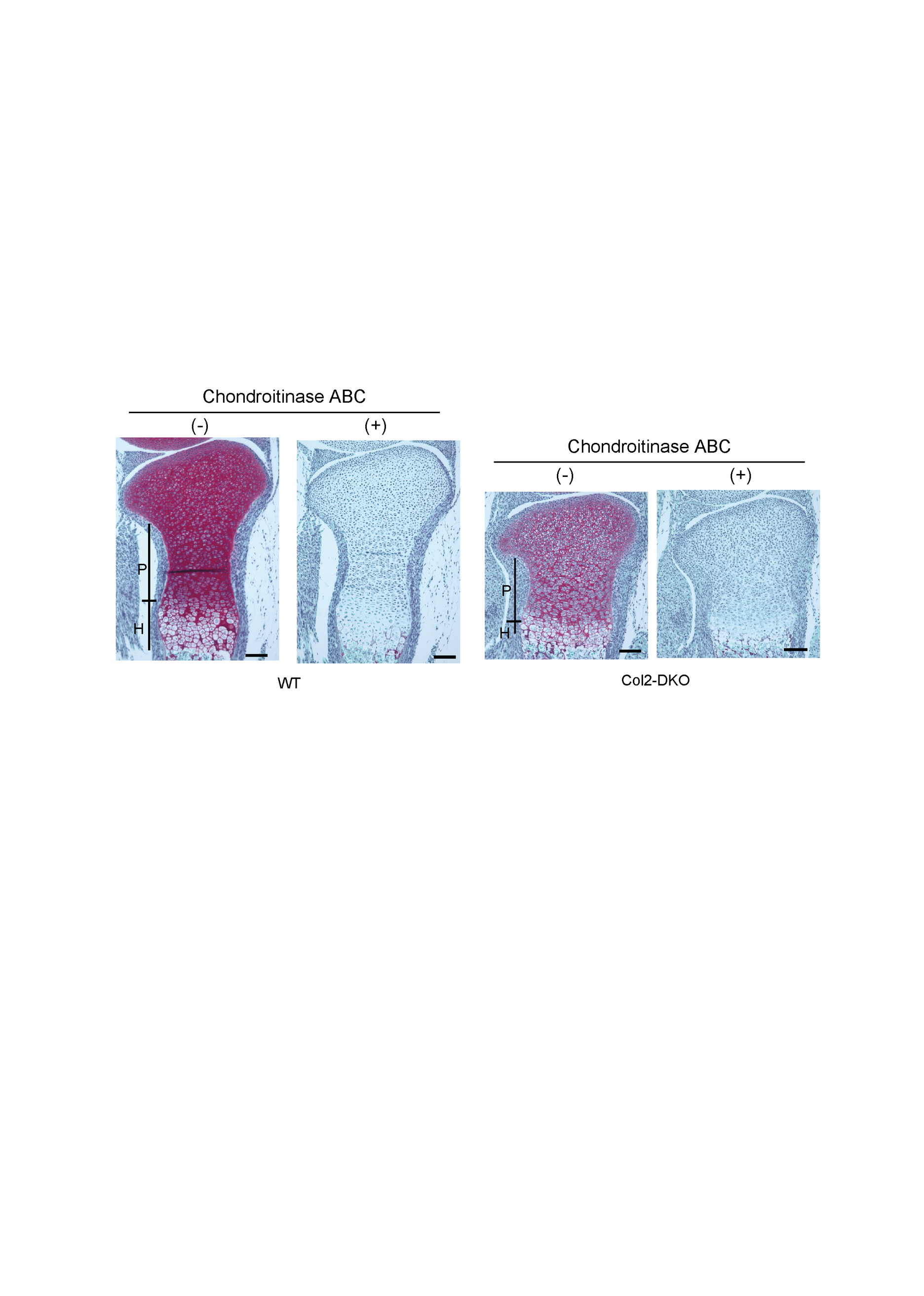

Supplement: S2 Fig — (TIF) [file pone.0190333.s002.tif]

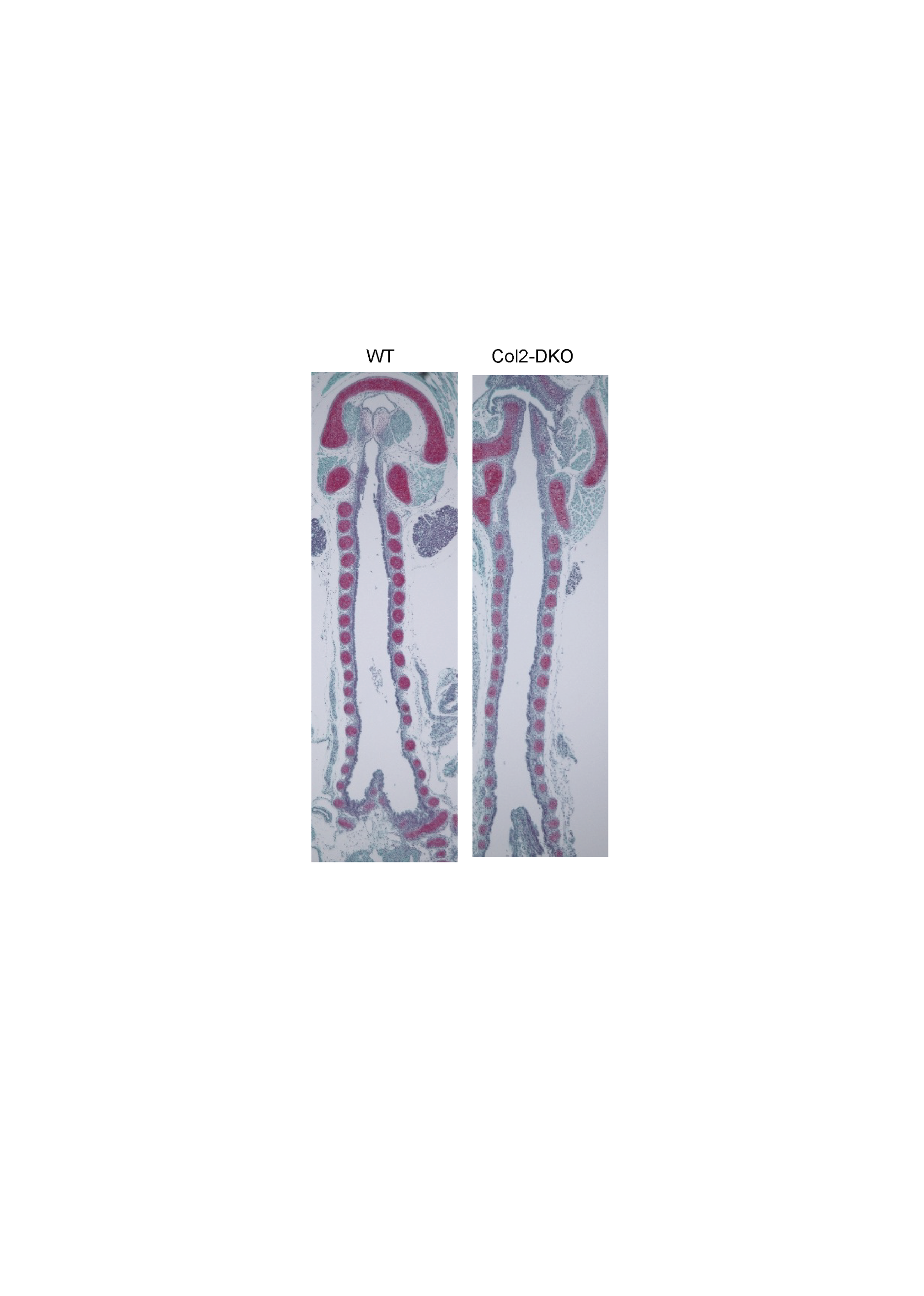

Supplement: S3 Fig — (TIF) [file pone.0190333.s003.tif]
